# Rapid Metagenomic Next-Generation Sequencing during an Investigation of Hospital-Acquired Human Parainfluenza Virus 3 Infections

**DOI:** 10.1128/JCM.01881-16

**Published:** 2016-12-28

**Authors:** Alexander L. Greninger, Danielle M. Zerr, Xuan Qin, Amanda L. Adler, Reigran Sampoleo, Jane M. Kuypers, Janet A. Englund, Keith R. Jerome

**Affiliations:** a Department of Laboratory Medicine, University of Washington, Seattle, Washington, USA; b Department of Pediatrics, University of Washington, Seattle, Washington, USA; c Vaccine and Infectious Disease Division, Fred Hutchinson Cancer Research Center, Seattle, Washington, USA; Boston Children's Hospital

**Keywords:** HPIV3, hospital-acquired infections, human parainfluenza 3 virus, infection prevention, mNGS, metagenomics, next-generation sequencing, parainfluenza, rapid sequencing, viral sequencing

## Abstract

Metagenomic next-generation sequencing (mNGS) is increasingly used for the unbiased detection of viruses, bacteria, fungi, and eukaryotic parasites in clinical samples. Whole-genome sequencing (WGS) of clinical bacterial isolates has been shown to inform hospital infection prevention practices, but this technology has not been utilized during potential respiratory virus outbreaks. Here, we report on the use of mNGS to inform the real-time infection prevention response to a cluster of hospital-acquired human parainfluenza 3 virus (HPIV3) infections at a children's hospital. Samples from 3 patients with hospital-acquired HPIV3 identified over a 12-day period on a general medical unit and 10 temporally associated samples from patients with community-acquired HPIV3 were analyzed. Our sample-to-sequencer time was <24 h, while our sample-to-answer turnaround time was <60 h with a hands-on time of approximately 6 h. Eight (2 cases and 6 controls) of 13 samples had sufficient sequencing coverage to yield the whole genome for HPIV3, while 10 (2 cases and 8 controls) of 13 samples gave partial genomes and all 13 samples had >1 read for HPIV3. Phylogenetic clustering revealed the presence of identical HPIV3 genomic sequence in the two of the cases with hospital-acquired infection, consistent with the concern for recent transmission within the medical unit. Adequate sequence coverage was not recovered for the third case. This work demonstrates the promise of mNGS for providing rapid information for infection prevention in addition to microbial detection.

## INTRODUCTION

Healthcare-associated infections (HAIs) affect hundreds of millions of patients around the world and cost hospitals tens of billions of dollars each year (1). Hospital-acquired viral infections are a particular problem in pediatric settings, with upwards of one-third of HAIs attributable to viruses (2). Hospital acquisition of human parainfluenza viruses (HPIVs) has been recognized as a particular problem among patients with cancer, including pediatric patients (3). In one study, 80% of HPIV infections in pediatric cancer patients were acquired in the hospital (4).

Recently, next-generation sequencing of pathogens from clinical samples has been used to inform infection prevention, to identify antimicrobial resistance, and to broaden differential diagnoses (5–10). Whole-genome sequencing (WGS) of clinical samples has been most commonly used for the genomic epidemiology of bacteria in hospital-acquired infections, where culture isolates are readily attainable, are part of the normal workflow, and undergo minimal mutation during the culture process (11–13). WGS for viral pathogens is considered more difficult given the lack of routine culture and propensity of viral culturing to induce mutations in viral genomes (7, 14). We used metagenomic next-generation sequencing (mNGS) for rapid sequencing of whole genomes from a putative cluster of human parainfluenza 3 virus (HPIV3) to determine whether a common source was on the medical unit. This is the first reported use of metagenomic next-generation sequencing in real time to inform hospital infection prevention.

### Outbreak description.

In June 2016, routine surveillance revealed three contemporaneous cases of hospital-acquired HPIV3 infection on a general medical unit. The first case was an 18-month-old male patient with chronic lung disease who developed increased work of breathing on day 109 of hospitalization. The patient required increasing oxygen support and was admitted to the pediatric intensive care unit due to worsening hypoxemia. The patient was managed with a short course of albuterol and steroids, and he recovered. Three days later, a second case was identified. Patient 2 was an 8-month-old boy with chronic lung disease who developed increased work of breathing on day 98 of hospitalization. This patient was also managed with a short course of albuterol and steroids and recovered. Then, 12 days after the first case, the third case was identified. Patient 3, a 4-month-old boy with congenital immunodeficiency, developed a cough and rhinorrhea on day 22 of hospitalization. The patient's symptoms resolved spontaneously without any interventions. Nasal swabs from all 3 patients tested positive for HPIV3 by real-time (RT)-PCR.

### Outbreak investigation.

In response to the cluster, the medical unit leadership and hospital infection prevention department conducted an investigation to identify potential sources of infection. Information about patient room locations, nursing assignments, involved provider teams, support services received by the patient, shared equipment, and patient movement in the hospital (to radiology, etc.) was collected. In addition, family members of patients and health care workers were queried about recent illness. The investigation identified that a health care worker with an upper respiratory tract infection had provided care to two of the health care-associated cases (patients 1 and 2).

In response to the event, nursing and physician staff received notification about the infections and education about the transmission of HPIV3. They were also reminded to stay home when ill. This information was included in weekly newsletters and daily unit-based huddles.

Respiratory samples obtained from the 3 putative hospital-acquired HPIV3 cases and 10 control patients were sent to the University of Washington virology laboratory for mNGS 2 days after recognition of the cluster (18 days after the first patient was diagnosed with HPIV3). The original sample for the third outbreak patient was no longer available, so a second sample taken 6 days later was used for sequencing.

## RESULTS AND DISCUSSION

### Case patients.

The cluster of 3 male patients with hospital-acquired HPIV3 ranged in age from 4 to 18 months. Additionally, all case patients had underlying medical conditions requiring frequent or prolonged hospitalization. None of the case patients had recently traveled.

### Control patients.

The 10 outpatients with community-acquired HPIV3 identified to serve as controls tested positive for HPIV3 by PCR within 15 days of the first case patient. Control patients were a median age of 9 months (range, 2 weeks to 7 years) and 60% were male. Additionally, 60% of the control patients were previously healthy and 40% were hospitalized for respiratory symptoms. One of the control patients (patient 9) had recently traveled to the Midwest. All other patients came from western Washington state.

### Metagenomic sequencing revealed outbreak strain.

**FIG 1:**
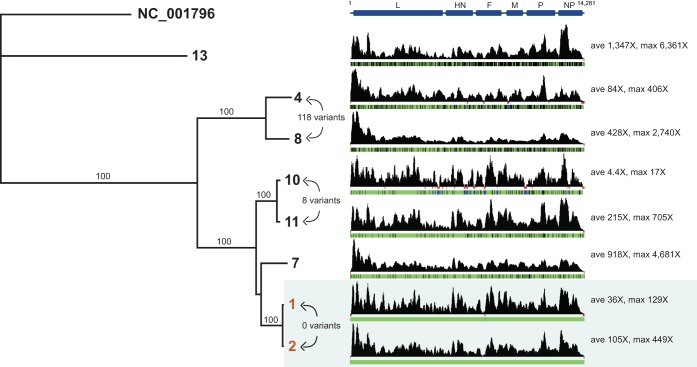
Whole-genome phylogeny of HPIV3 sequences revealed identical sequences in putative outbreak samples. Near-complete genomes recovered from HPIV3-positive specimens were aligned by MAFFT, and phylogenetic trees were constructed using MrBayes. Specimens are denoted by sample identifier name with the two identical clinical cases highlighted in orange. The NCBI reference genome for HPIV3 (NC_0001796) was used as an outgroup and its negative-stranded genome is highlighted in green. Consensus support values are denoted on branch labels. Normalized coverage maps are shown with areas of no coverage underlined in red and nucleotide changes relative to the outbreak sequence highlighted in black. Pairwise variant counts are depicted for three of the most closely related pairs of strains sequenced.

A total of 22,904,287 quality/adapter-trimmed reads were derived from the 13 samples with a median trimmed read count of 1,666,529 reads per sample. The number of HPIV3 reads in each sample ranged from 4 reads to 202,946 reads (median, 5,823 reads) (Table 1). A total of 8 of the 13 samples (2 cases and 6 controls) had sufficient coverage to yield near-whole genomes for phylogenetic analysis. Phylogenetic analysis revealed that the 8 samples came from 6 distinct lineages, with the two hospital-acquired strains sharing the exact same sequence across the entire genome and not identical to any of the control strains (Figure 1). Extending the tree to include 10 partial genomes from all samples with >50 reads mapping to HPIV3 (2 cases and 8 controls) showed 9 strains (see Fig. S1 in the supplemental material). The third sample from a hospital-acquired case had 4 reads, each of which had a sequence identical to those of the genomes recovered from the 2 outbreak strains (see Fig. S2). Retrospective quantitative RT-PCR revealed a linear relationship between percent reads to HPIV3 recovered with the approximate copies/ml of inoculated viral transport media, with patient 7 the one outlier among the 13 samples (Table 1).

**TABLE 1:** Specimens sequenced in this study

Origin and patient no.	Time (d) determined positive compared to index case	Age	Sex	Copies/ml	No. of reads	
					Filtered	HPIV3
Hospital acquired						
1	0	18 mo	Male	1.90 × 10^7^	1,814,866	5,823
2	3	8 mo	Male	2.85 × 10^8^	1,864,624	16,901
3	12	4 mo	Male	6.40 × 10^4^	1,097,618	4
Community acquired						
4	−8	2 yr	Male	1.51 × 10^6^	1,286,425	11,605
5	−2	2 mo	Female	5.59 × 10^5^	1,960,840	21
6	1	1 yr	Male	1.02 × 10^4^	1,444,230	4
7	2	9 mo	Male	1.62 × 10^8^	1,565,065	132,401
8	3	2 wk	Female	1.19 × 10^9^	1,573,141	54,369
9	8	7 yr	Female	3.88 × 10^3^	1,054,320	88
10	10	22 mo	Female	6.67 × 10^6^	1,956,632	631
11	11	1 mo	Male	2.11 × 10^8^	1,666,529	31,502
12	13	2 mo	Male	6.28 × 10^6^	2,223,697	356
13	15	1 yr	Male	3.13 × 10^9^	3,396,300	202,946

BLASTN analysis of the outbreak strain revealed 100% nucleotide identities to hemagglutinin-neuraminidase (HN) genes from a 2011 Argentinian strain HNRG.036.11 (1,146 bp; KT765972), a March 2010 Colombia strain FLI2812 (352 bp; JQ268865), and a 2012 Japan strain 70-PIV3 (147 bp; AB831638). The closest whole-genome sequence was from the October 2007 FLU8652 strain from Piura, Peru. BLASTN analysis of the farthest outgroup strain (patient 13) revealed 99.8% nucleotide identity to HN genes from the 1995 nosocomial outbreak strain from Massachusetts (427 bp; AF039925) and 98.3% nucleotide identity to the whole-genome sequence from the HPIV3 cell culture strain 14702 originally isolated from Canada (15,462 bp; EU424062).

Bayesian analysis revealed an average 7.61 × 10^−4^ nucleotide substitutions per site per year across the HPIV1 genome (with 1.37 × 10^−3^ substitutions in the HN gene) (15). An analysis of HPIV3 strains in Japan showed approximately the same rate of evolution in the HN gene for HPIV3 (16). These data indicate that approximately 11 sites change per year across the HPIV3 genome, suggesting the resolution of one nucleotide change in the HPIV3 genome in terms of transmission is approximately 1 month.

Here we report the first rapid use of metagenomics to inform infection prevention in a hospital setting. Previous approaches using next-generation sequencing to inform infection control were entirely retrospective, were focused on pure bacterial isolates, and/or utilized PCR tiling approaches based on a known organism (12, 13, 17–22). In this study, mNGS retrieved identical whole-genome HPIV3 sequences from two patients, confirming an association between the two cases and suggesting transmission from a single source. mNGS also recovered reads from a third outbreak patient that had identical sequences to the two patients in the cluster, but the read coverage was not sufficient to determine with confidence that this case arose from the same source. Metagenomic sequencing was performed with a rapid turnaround time (<3 days) with approximately 6 to 8 h of hands-on time. Linked bioinformatics analyses might perform the same task with sample-to-answer in under 30 h based on currently available Illumina sequencers and the depth of sequencing required. Nanopore sequencing might reduce this turnaround time even more; however, this method is not amenable to mNGS due to the limited depth of sequencing (17, 23).

The main limitation of the approach taken here is the reduced sensitivity of mNGS relative to that of PCR (24). mNGS is often unable to recover whole genomes from FilmArray-positive specimens, as illustrated in this study. In this study, near-complete genomes were recovered from only 8 of 13 specimens with a median of 1.7 million trimmed reads per sample, while partial genomes were recovered from 10 of 13 samples. One of the specimens for which a whole-genome HPIV3 sequence could not be retrieved was part of the putative cluster, reducing the ability to confidently link further transmission within the hospital. Of note, this specimen was obtained via a repeat swab approximately 6 days after the patient's first positive test for HPIV3, and thus it is not surprising that little sequence could be recovered. In addition, whole genomes are not required for linking cases, as phylogenetic information can be recovered with only partial sequences (25).

A definitive advantage of mNGS is the use of only one protocol for detection and genome-wide analysis of a myriad of infectious diseases, which can aid in personnel training and reduce turnaround time relative to other genome portioning methods. A potential limitation of this approach is cost, as we estimated that the marginal reagent cost of this sequencing was approximately $2,000. However, the cost for mNGS versus capture sequencing methods is approximately the same, and the temporal resolution and epidemiological information afforded by whole-genome methods greatly exceed that afforded by candidate gene sequencing (26).

Molecular tools enable the investigation of infection clusters and outbreaks, both in community and hospital settings. mNGS adds to the available tools and enables unbiased pan-pathogen detection with single-nucleotide resolution, which enables detection and inference of transmission. In the hospital setting, this molecular data can play a critically important role in convincing health care workers and administrators that transmission is occurring and can provide a rationale for expending resources and targeting interventions to prevent further transmission. The provision of whole-genome data in a rapid and actionable time frame that advances clinical care will continue to be one of the major challenges as this technology moves from a research to a clinical setting.

## MATERIALS AND METHODS

### Setting.

Seattle Children's Hospital is a 316-bed quaternary care pediatric facility located in Seattle, Washington. The medical unit involved is located on one floor and includes 32 beds. Patients housed on the unit are newborn to 21 years of age and have a wide variety of acute and chronic health issues. Respiratory viral testing is routinely performed on symptomatic patients using nasal swabs that are evaluated using a FilmArray assay (BioFire, Salt Lake City, UT).

### Ethical concerns.

The study protocol was approved by the Children's institutional review board. Consent from patients was deemed unnecessary as all data already existed and were made anonymous for the purpose of this study.

### Cluster and control samples.

Cluster samples were clinical samples that were positive for HPIV3 by PCR from a patient hospitalized on the general medical unit who met criteria for hospital-acquired HPIV3 (clinical symptoms that developed >6 days after admission). These criteria were based on the incubation period of HPIV (27).

For strain comparison, clinical microbiology records were used for identifying a convenience sample of control samples from inpatient and outpatient children testing positive for HPIV3 by RT-PCR that were obtained during the same month as the cluster from patients with community-acquired HPIV3.

### mNGS library generation and sequencing.

A 500-μl volume of nasal swab-inoculated viral transport medium was filtered through a 0.45 μm Ultrafree-MC spin filter (Millipore), and 200 μl of filtrate was used as input for extraction in a ZR viral RNA kit (Zymo Research). Extracted RNA was treated with Turbo DNase (LifeTech) and first- and second-strand synthesis was performed using random hexamers and SuperScript III (Life Tech) and Sequenase (Agilent) enzymes (28). cDNA was purified using a DNA clean and concentrator-5 kit (Zymo) and used as input for Nextera XT library generation with 20 cycles of PCR amplification. Sequencing libraries were purified using 1.0× AMPure XP beads (Beckman Coulter), quantified on a Qubit 3.0 (LifeTech) and a Bioanalyzer 2100 (Agilent) (29). Samples were mixed in equimolar to achieve approximately 2 million reads per sample and sequenced using a 1×180 bp run on a MiSeq desktop sequencer (Illumina).

Sequencing reads were adapter- and quality-trimmed using cutadapt and mapped to the NCBI reference genome for HPIV3 (NC_001796) using Geneious v9.1 software (30). Consensus sequences of regions with >1× coverage were called by majority-voting base with hand curation. Genome alignments were made using MAFFT and phylogenetic trees were created using MrBayes (31, 32).

### Retrospective quantitative RT-PCR.

RNA from samples that were filtered and extracted for mNGS was tested by real-time RT-PCR for quantitation of HPIV3. HPIV3 RNA was detected using primers and probe that target the HPIV3 matrix gene, and the number of genome copies/ml was quantified using a standard curve generated by amplification of known numbers of RNA transcripts containing the HPIV3 RT-PCR amplicon sequences (33).

### Accession number(s).

HPIV3 genomes were deposited in GenBank under accession no. KX574704 to KX574711.

## Supplementary Material

Supplemental material
