# Supplementary material for: Rapid Metagenomic Next-Generation Sequencing during an Investigation of Hospital-Acquired Human Parainfluenza Virus 3 Infections
Source: J Clin Microbiol. 2016 Dec 28;55(1):177–82. doi: 10.1128/JCM.01881-16 (PMC5228228; doi:10.1128/JCM.01881-16)
Supplement: Supplemental material [file supp_55_1_177__index.html]

Supplemental material 

# Rapid Metagenomic Next-Generation Sequencing during an Investigation of Hospital-Acquired Human Parainfluenza Virus 3 Infections

## Supplemental material

- Supplemental file 1 -

  Fig. S1 (Partial-genome phylogenetic analysis of samples with >50 HPIV3 reads reveals that additional community-acquired cases were not part of the hospital outbreak)

  PDF, 101K
- Supplemental file 2 -

  Fig. S2 (Phylogenetic analysis of most informative 99-bp trimmed read from patient 3 across all 10 samples for which sequence was available at that locus)

  PDF, 97K
