# Supplementary material for: Rapid Metagenomic Next-Generation Sequencing during an Investigation of Hospital-Acquired Human Parainfluenza Virus 3 Infections
Source: J Clin Microbiol. 2016 Dec 28;55(1):177–82. doi: 10.1128/JCM.01881-16 (PMC5228228; doi:10.1128/JCM.01881-16)
Supplement: Supplemental material [file JCM.01881-16_zjm999095305s1.pdf]

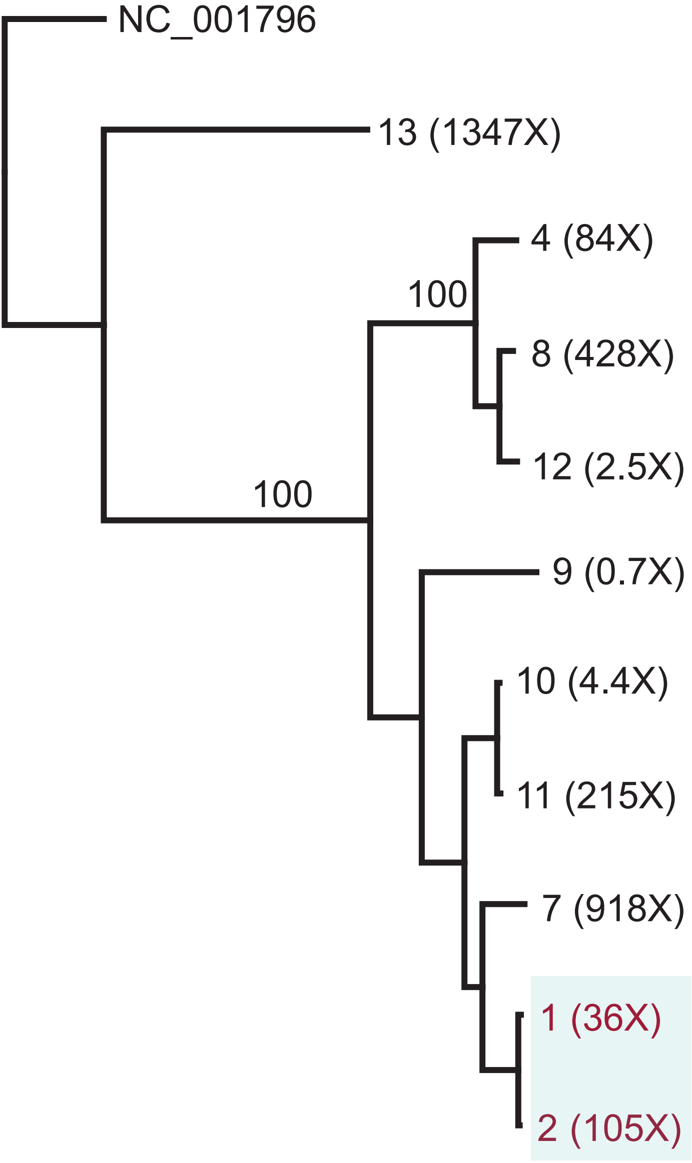

Supplemental Figure 1. Partial genome phylogenetic analysis of samples with >50 HPIV3 reads reveals that additional community-acquired cases were not part of the hospital outbreak.
