# Supplementary material for: Rapid Metagenomic Next-Generation Sequencing during an Investigation of Hospital-Acquired Human Parainfluenza Virus 3 Infections
Source: J Clin Microbiol. 2016 Dec 28;55(1):177–82. doi: 10.1128/JCM.01881-16 (PMC5228228; doi:10.1128/JCM.01881-16)
Supplement: Supplemental material [file JCM.01881-16_zjm999095305s2.pdf]

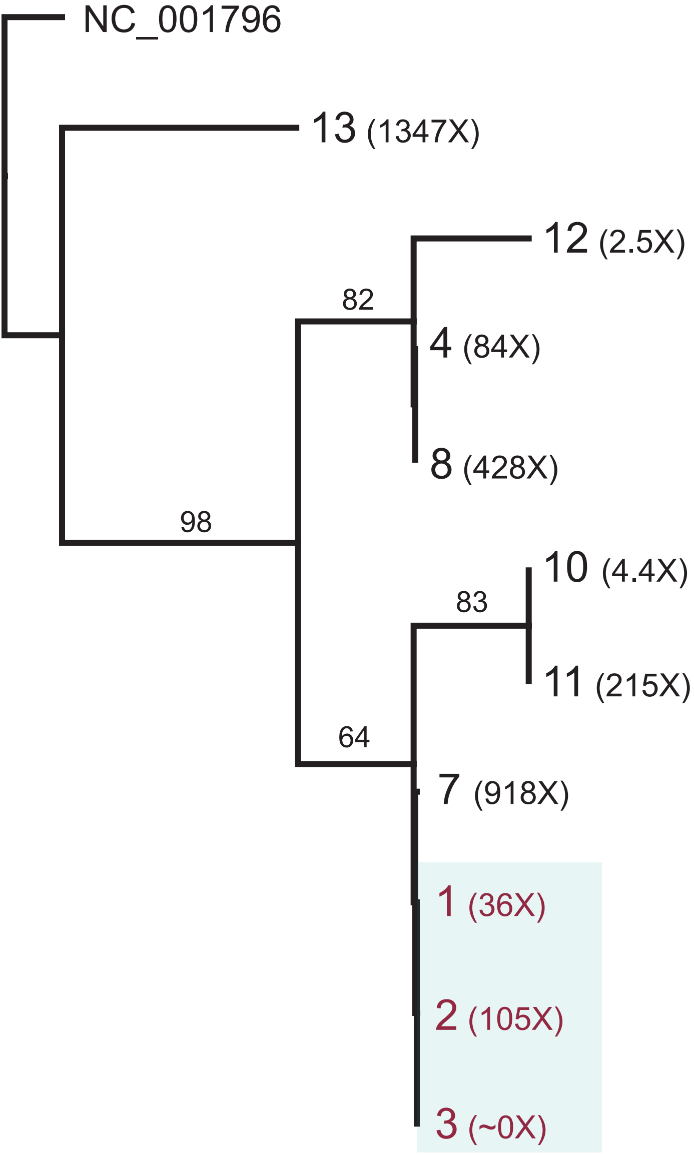

Supplemental Figure 2. Phylogenetic analysis of most informative 99bp trimmed read from patient 3 across all 10 samples for which sequence was available at that locus. Patient 3 clusters within the hospital outbreak;
however, this limited sequence is not present in all low-coverage samples.
